# Combining morpho-taxonomy and metabarcoding enhances the detection of non-indigenous marine pests in biofouling communities

**DOI:** 10.1038/s41598-018-34541-1

**Published:** 2018-11-02

**Authors:** Ulla von Ammon, Susanna A. Wood, Olivier Laroche, Anastasija Zaiko, Leigh Tait, Shane Lavery, Graeme J. Inglis, Xavier Pochon

**Affiliations:** 1 Environmental Technologies, Coastal and Freshwater Group, Cawthron Institute, Private Bag 2, Nelson, 7042 New Zealand; 2 School of Biological Sciences, University of Auckland, Private Bag 92019, Auckland, 1142 New Zealand; 3 National Institute of Water & Atmospheric Research Ltd, PO Box 8602, Riccarton, Christchurch 8440 New Zealand; 4 Institute of Marine Science, University of Auckland, Private Bag 349, Warkworth, 0941 New Zealand

## Abstract

Marine infrastructure can favor the spread of non-indigenous marine biofouling species by providing a suitable habitat for them to proliferate. Cryptic organisms or those in early life stages can be difficult to distinguish by conventional morphological taxonomy. Molecular tools, such as metabarcoding, may improve their detection. In this study, the ability of morpho-taxonomy and metabarcoding (18S rRNA and COI) using three reference databases (PR2, BOLD and NCBI) to characterize biodiversity and detect non-indigenous species (NIS) in biofouling was compared on 60 passive samplers deployed over summer and winter in a New Zealand marina. Highest resolution of metazoan taxa was identified using 18S rRNA assigned to PR2. There were higher assignment rates to NCBI reference sequences, but poorer taxonomic identification. Using all methods, 48 potential NIS were identified. Metabarcoding detected the largest proportion of those NIS: 77% via 18S rRNA/PR2 and NCBI and 35% via COI/BOLD and NCBI. Morpho-taxonomy detected an additional 14% of all identified NIS comprising mainly of bryozoan taxa. The data highlight several on-going challenges, including: differential marker resolution, primer biases, incomplete sequence reference databases, and variations in bioinformatic pipelines. Combining morpho-taxonomy and molecular analysis methods will likely enhance the detection of NIS from complex biofouling.

## Introduction

Biological invasions of non-indigenous species (NIS) can cause severe economic and environmental impact contributing to biodiversity loss^1,2^. One of the major vectors responsible for the transfer of marine NIS is global shipping via ballast water^3^ and hull fouling^4^. The ports and marinas where these vessels berth often act as hubs for the spread of NIS^5,6^. Artificial substrata such as wharf piles and pontoons, which might be less attractive for native taxa, provide opportunistic NIS with vacant niches where they can settle and establish thriving populations^6,7^.

Early detection of NIS is a critical factor to inform timely implementation of measures and allow the greatest chance of successful management^1^. Current marine surveillance programmes largely rely on traditional morphological identification of NIS during visual surveys by divers and from biological samples collected by a variety of methods, including grabs, benthic sleds, trawls, and passive sampling devices such as settlement plates^8–10^. Morpho-taxonomy is particularly well suited for conspicuous organisms, such as macrofauna or macroalgae that can be readily identified. However, the identification of cryptic or small juvenile life stages *in situ* or within complex samples remains challenging^11,12^.

Recent advances in molecular technologies, in particular the emergence of high-throughput sequencing (HTS), are rapidly changing the way biomonitoring programmes are undertaken^13^. For example, metabarcoding is increasingly being used for biodiversity assessments from a range of aquatic ecosystems^14–20^. The potential for high sensitivity, accuracy and standardizing and automating these methods make metabarcoding a particularly well-suited approach for use in marine biosecurity surveillance programmes^21–25^.

Nonetheless, metabarcoding approaches have limitations^3,14,26,27^. For example, databases of reference DNA sequences are incomplete^28^ and are often tailored for certain genetic markers or taxonomic groups (e.g. Protist Ribosomal Reference [PR2] database for the nuclear small ribosomal subunit 18S rRNA)^29^. GenBank^30^ contains reference sequences from many different genetic markers and includes all domains of life but is prone to erroneously identified sequences^31^. Another limitation of metabarcoding is a lack of ‘universal’ markers across phyla^5^, therefore not all taxa can be equally detected due to primer selectivity and resulting amplification biases^32^. Recent studies have highlighted the advantage of using multiple barcode regions, including mitochondrial *Cytochrome oxidase c subunit 1* (COI) for discriminating between many metazoan species, and 18S rRNA that enables the detection of a much broader range of taxa^5,33,34^.

Appropriate bioinformatic pipelines can improve the robustness of molecular biodiversity assessment, and derived taxonomic assignments may vary based on the parameters applied^35^. One of the critical steps in a pipeline is the grouping of similar sequences into Operational Taxonomic Units (OTUs), to count for intraspecific variation within taxa and sequencing errors. Although a 97% similarity threshold is commonly applied for general biodiversity assessments, a higher threshold may increase rare taxa detection, which may be particularly well suited when targeting NIS^36^.

Combining the use of conventional morpho-taxonomic and metabarcoding approaches may enhance detection sensitivity^37^ and lead to the minimization of Type-I errors (false positives) and Type-II errors (false negatives) in marine NIS surveillance programmes^2^. In this study, an integrated morpho-taxonomic and metabarcoding approach was used to characterize biodiversity on settlement plates, with a focus on the detection of NIS within the samples. The overall goal was to investigate the benefits of integrating molecular methods such as metabarcoding into conventional marine NIS surveillance programmes to collectively enhance the likelihood of early detections. The metabarcoding approach comprised two molecular marker regions, the V4 region of 18S rRNA and a small region of COI. Taxonomy was assigned against three sequence databases (The Protist Ribosomal Reference database [PR2], The Barcode of Life Database [BOLD] and the nucleotide collection of the National Centre for Biotechnology Information [NCBI]). The following hypotheses were tested: that biofouling diversity and NIS detection would differ significantly (1) between morphological and molecular approaches due to varying efficiency in identifying micro-, meio- and macrofaunal communities, and (2) between molecular markers (18S rRNA and COI) due to differences in the taxonomic resolution and coverage of sequence reference databases.

## Material and Methods

### Experimental design

This research was implemented as a companion study to a project that used morpho-taxonomic identification to investigate the optimal design of settlement arrays for sampling non-indigenous biofouling species^10,38^. Five settlement arrays each consisting of twelve polyvinyl chloride (PVC) settlement plates (14.5 cm × 14.5 cm) attached to PVC pipes were deployed at randomly chosen pontoons in Westhaven Marina, Auckland, New Zealand. Separate deployments were made in winter (June to October 2015) and summer (November 2015 to February 2016) months. Each settlement array was placed horizontally at two meters depth and comprised of three different treatments in a crossed experimental design. The design included assessing the effect of different antifouling coatings, light intensities and surface textures on NIS detection as previously described in von Ammon, *et al*.^39^ and Tait, *et al*.^38^. The present study does not address the effect of these treatments but considers combined data from all five arrays. Each plate was considered as an individual sample while most downstream analyses (apart from rarefaction curves) were processed on combined data of all 60 samples for summer and winter each.

### Morpho-taxonomic approach

All plates were retrieved after three-month deployment periods and immediately transported to the laboratory on ice and in individual bags filled with seawater. Visible taxa were identified and species richness was determined under a light microscope at 10x magnification as in Tait, *et al*.^38^. Abundant species from the upper layer of biofouling were partially removed to identify biofouling organisms in secondary cover. Voucher specimens of unknown species were preserved in ethanol and sent to taxonomic specialists for identification. During this handling, care was taken to ensure that a portion of each morphologically identified taxon remained on the plate for subsequent metabarcoding analyses. All specimens were identified to the lowest possible taxonomic level, and categorized as either indigenous to New Zealand, non-indigenous, cryptogenic (undetermined geographic origin), or unresolved. Unresolved individuals accounted for less than 10% of taxa and were excluded from downstream NIS analyses, as their biosecurity status could not be determined.

### Metabarcoding approach

Following the morpho-taxonomic screening, the five identical arrays (*n* = 60 plates) were sampled for metabarcoding analysis. All biofouling material was removed from the plate surface using sterile stainless steel surgical blades (Swann-Morton^R^, Sheffield, UK) and transferred into a 10 mL sterile tube (Merck KGaA, Darmstadt, Germany) in February (summer) or sterilized sponges (Whirl-pak™, Speci-sponges™, Nasco, WI, USA) stored in individual sterile plastic bags in October (winter). All samples were stored immediately at −70 °C until further processing. The sampling technique varied between summer and winter samples due to the high amounts of calcifying organisms on the summer plates which could not be removed with the sponge method used for winter samples.

The summer samples were centrifuged (4000 × g, 15 min) and the supernatant discarded. The winter samples (sponges with biofilm samples) were macerated using a stomacher (Colworth 400; AJ Seward, London, UK) for 2 min at maximum speed, then squeezed to remove excess liquid. The resulting biofouling suspensions were pelleted by centrifugation (4000 × g, 15 min) and the supernatant discarded. DNA was extracted from the resulting pellets using the PowerMax® Soil DNA Isolation Kit (QIAGEN, CA, USA) following the manufacturer’s protocol. The quantity and quality of extracted DNA were measured using a NanoPhotometer (Implen, Munich, Germany).

For the characterization of eukaryotic communities, a segment (approximately 400 base pairs [bp]) of the V4 region of the 18S rRNA gene and an approximately 300 bp fragment of the mitochondrial COI gene were amplified by Polymerase Chain Reaction (PCR). For the 18S rRNA gene, the eukaryotic-specific primers were Uni18SF: 5′-AGG GCA AKY CTG GTG CCA GC-3′ and Uni18SR: 5′-GRC GGT ATC TRA TCG YCT T-3′^40^ modified to include Illumina^TM^ overhang adaptors following von Ammon, *et al*.^39^. Thermocycling PCR conditions were: 94 °C for 3 min followed by 35 cycles of 94 °C for 30 s, 52 °C for 30 s, 72 °C for 1 min, with a final extension step at 72 °C for 7 min. For the COI gene, the eukaryotic-specific primers mlCOIintF: 5′-GGW ACW GGW TGA ACW GTW TAY CCY CC-3′ and jgHCO2198: 5′-TAI ACY TCI GGR TGI CCR AAR AAY CA-3′ were used^41^. PCR amplification was undertaken in a total volume of 50 μL using 25 μL of MyTaq™ Red Mix (Bioline, USA), 2 μL of each primer, 16 μL of DNA-free water, 3 μL of BSA (0.2 mg/mL) and 2 μL of template DNA. Thermocycling conditions were: 95 °C for 3 min, followed by 40 cycles of 95 °C for 30 s, 50 °C for 30 s, 72 °C for 90 s, and a final extension of 72 °C for 10 min. Two samples of 20 μL of ddH_2_O were used as negative controls following the same protocol as described above.

Purification and quantification of amplicons were performed following the Agencourt® AMPure® XP protocol (Beckman Coulter, USA), using magnetic beads and a Qubit® 2.0 Fluorometer (Invitrogen). Purified amplicons were diluted to 3 ng μL^−1^ and libraries sent to New Zealand Genomics Limited at the University of Auckland for sequencing. Paired-end sequences (2 × 250) were generated on a MiSeq instrument using the TruSeq^TM^ SBS kit v3 (Illumina^TM^).

### Bioinformatics analyses

The raw sequence files were demultiplexed using fastq-multx (version 1.3.1) and bi-directional reads were paired using SolexaQA++^42^. By running the following pipeline on QIIME 1^43^, the reads were truncated on the 3′ end from the first base where the Phred score dropped below 3 and which explains, along with differences in sequence length among taxa, the variation in the overlap size and when paired-end reads were assembled. For quality filtering, merging and dereplication, the software package VSEARCH was used^44^. Merged reads with more than five bp expected error were discarded. Sequence chimeras were detected using VSEARCH Uchime de novo method by mapping unique 18S rRNA sequences against the PR2 database^29^ and COI sequences against the combined MIDORI^45^ and Barcode Of Life Database (BOLD; Ratnasingham and Hebert^46^) that were trimmed using the COI primers of this study. Sequence reads were clustered into OTUs at 99% similarity to retain maximum sensitivity for NIS detection. OTUs found in negative controls were discarded across all samples. Singletons and all low read OTUs were kept. Taxonomy was assigned using the QIIME package^43^ and the default UCLUST classifier with 0.9 minimum sequence identity^47^, with the PR2 and BOLD databases for 18S rRNA and COI clusters, respectively.

For cross-validation, both 18S rRNA and COI sequence datasets were also aligned against NCBI’s nucleotide collection (nr/nt) database (NCBI) using Megablast^48^, searching for a maximum of 10 best-matching sequences (Megablast+, default e-value of 0.001, word size 28). Using hits with the lowest e-value, query sequences were assigned at species level if similarity of the hit was greater or equal to 97%. Otherwise, if sequences did not fulfill the 97% or above threshold, the naïve Last Common Ancestor (LCA) and default parameters in MEGAN v.5^49^ among the best hits was used for assignment to higher taxonomic ranks. This process separates reads that align specifically to a single taxon and assigns these together and less specific reads that were aligned to different taxonomies by the 10 best matching hits in Megablast. In the latter case, if less than 75% of the hits share the same taxonomy, no assignment is made at this specific taxonomic rank and the algorithm repeats the process to the next level.

Sequences for the COI region have been deposited in the NCBI’s Sequence Read Archive under BioProject ID PRJNA478269, sample accession SAMN09508561–679 and for the 18S rRNA region in the European Nucleotide Archive (ENA) under BioProject PRJEB25036, sample accession SAMEA104599381-499.

### Statistical analyses

The phyloseq R package and associated tools^50^ were used for the following diversity analyses. Read abundance of metabarcoding data was rarefied to 5,000 reads. Ten samples were below this threshold and were discarded for the diversity analysis. The datasets were then filtered for Eukaryotes and merged at a genus level to be consistent with the morpho-taxonomic data and presence/absence transformed. Overall taxonomic richness (genus level) of samples from each dataset was visualized using boxplots.

Unrarefied OTU tables were filtered for Metazoa, Chlorophyta, Ochrophyta and Rhodophyta (taxonomic groups commonly identified in biofouling by morpho-taxonomic analysis). The abundance data was merged at the genus level. Bar plots for each approach were computed using standardized data from most abundant taxa.

A general list of NIS for New Zealand was assembled by consulting the Marine Invasive Taxonomic Service^51^ and through comparison with the New Zealand Organisms Register^52^ and the Marine Biosecurity Porthole^51^. This list was used to filter non-indigenous taxa on genus and species levels from the metabarcoding and morpho-taxonomic datasets. Euler diagrams were constructed on the filtered data using the online Venn diagram creator at http://bioinformatics.psb.ugent.be/webtools/Venn/ to quantify overlaps in NIS detected using the different approaches and proportionally visualized using the R package ‘eulerr’^53^. For assessing performance of NIS detections, rarefaction curves were computed on the filtered datasets of all approaches combining the information of NIS from PR2/NCBI for 18S rRNA and BOLD/NCBI for COI and their combinations using the iNEXT R package^54,55^.

## Results

### Morpho-taxonomy

**Figure 1:**
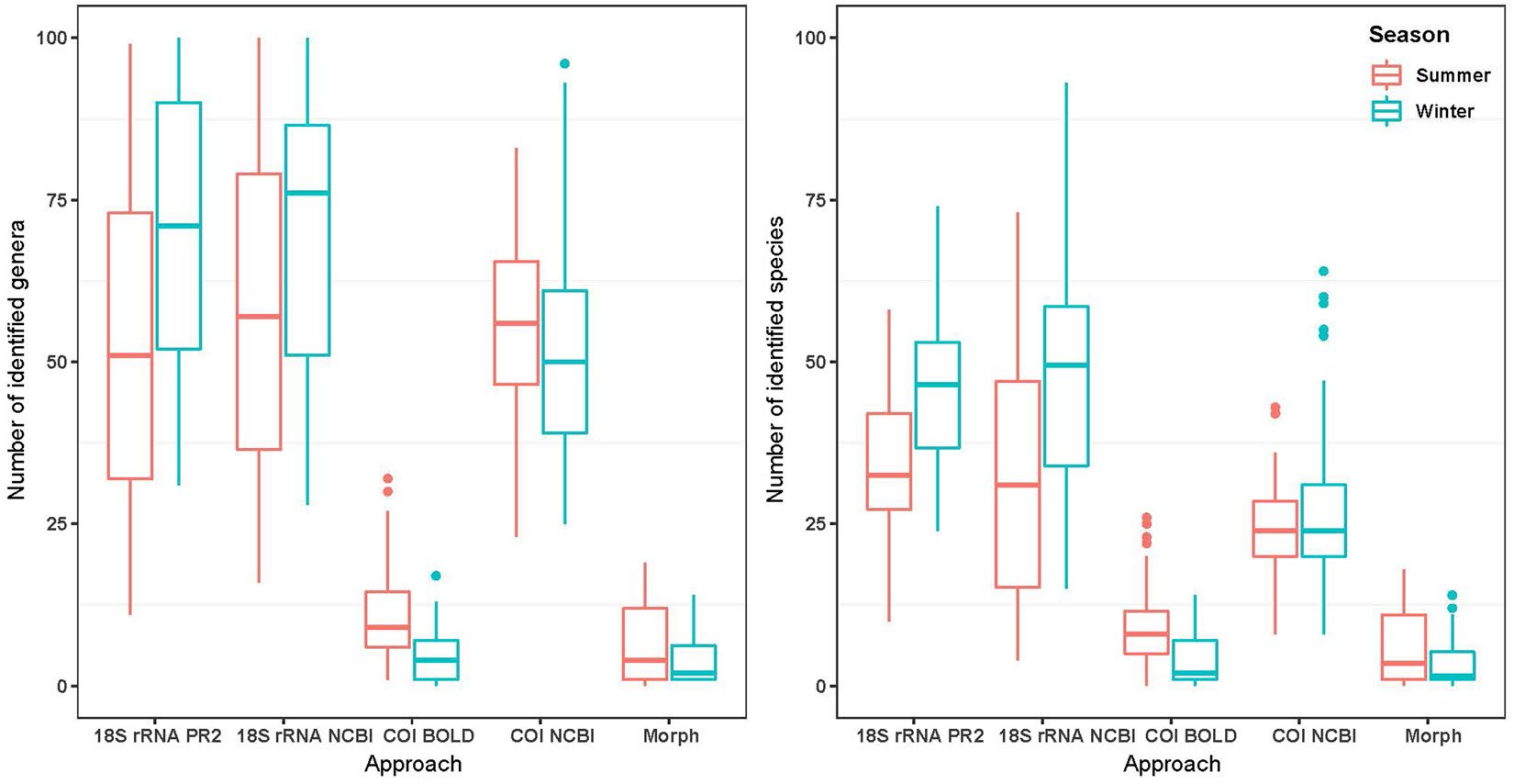
Boxplots displaying number of eukaryotic genera (left) and species (right) detected per summer and winter sample plates, using 18S ribosomal RNA (18S rRNA) and *Cytochrome c oxidase Subunit I* (COI) metabarcoding assigned to the three databases PR2 (18S rRNA), BOLD (COI) and NCBI (both 18S rRNA and COI) and morpho-taxonomy (morph). Molecular datasets were rarefied to 5,000 reads, with all genus-level unassigned sequences removed.

**Figure 2:**
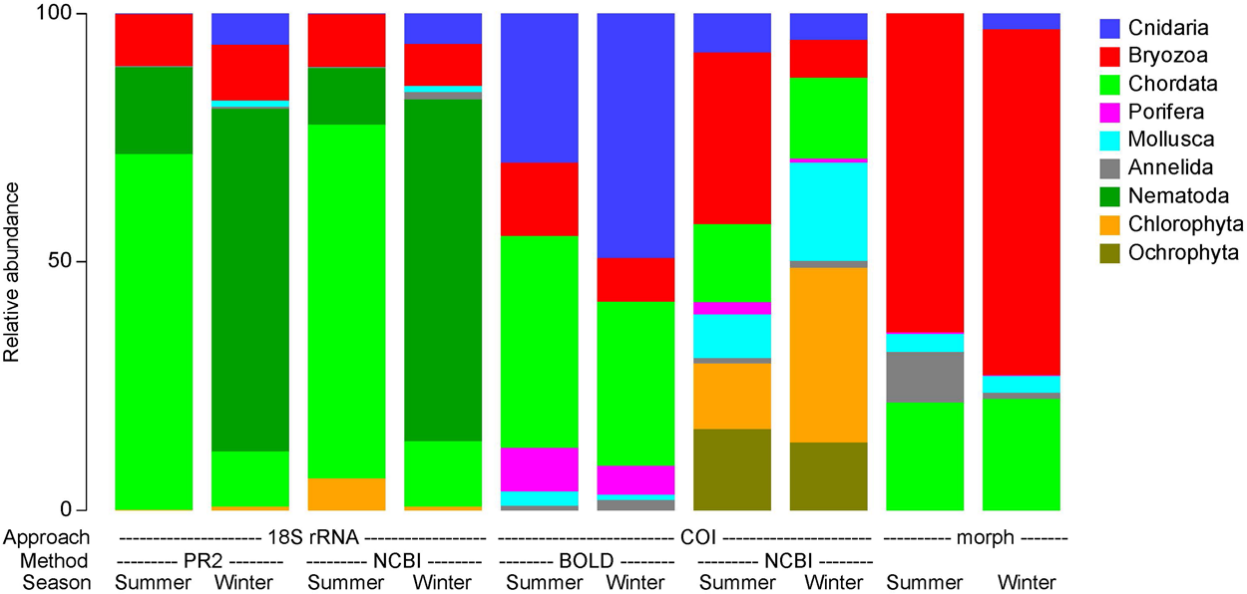
OTU proportions of the most abundant metazoan and algal taxa, standardized for all datasets ignoring the unassigned fraction: 18S ribosomal RNA (18S rRNA) and *Cytochrome c oxidase Subunit I* (COI) metabarcoding (PR2, BOLD and NCBI database assignments) and morphological taxonomy (morph) for summer and winter samples.

The morpho-taxonomic assessment resulted in the total observation of 39 taxa in summer (average 6.4 per sample plate) and 31 taxa in winter (4.2 per sample) of which 35 and 27, respectively, could be identified down to at least genus level, and 33 and 24 to species level (Fig. 1). The main phyla present were Bryozoa, Chordata, Mollusca and Annelida. For winter samples, Annelida were less abundant while Cnidaria increased in abundance (Fig. 2).

### Metabarcoding

The total paired-end, quality filtered and non-chimeric and de novo non-chimeric sequences obtained from combined summer and winter plates for 18S rRNA and COI PCR amplicons were 2,831,859 reads (95,751 unique OTUs) and 3,084,751 reads (211,164 unique OTUs), respectively (Table 1). Negative controls contained 41 reads assigned to 19 OTUs for 18S rRNA and COI, indicating insignificant background contamination, from which no new potential NIS were identified.

**Table 1:** Bioinformatic pipeline results, for 18S ribosomal RNA (18S rRNA) and *Cytochrome c oxidase Subunit I* (COI) across taxonomic assignment methods. In the de novo OTU picking process (*) reads are clustered against one another without any external reference sequence collection.

Approach (target gene)				
	**18S rRNA**		**COI**	
Raw sequences	15,235,416		10,638,458	
Merged reads	6,604,463		3,870,763	
Quality filtered	4,792,948		3,819,858	
Reference chimeras	65,497		671	
De novo* chimeras	68,230		57,700	
Non-chimeras	1,417,296		1,570,890	
De novo* non-chimeras	1,414,563		1,513,861	
Singletons	70,202		155,522	
No. of OTUs (99% similarity)	95,751		211,164	
**Method (taxonomy assignment & database)**				
	**18S rRNA**		**COI**	
	**UCLUST -PR2**	**Megablast/ LCA -NCBI**	**UCLUST - BOLD**	**Megablast/LCA -NCBI**
Unassigned OTUs	7,839	2,203	198,138	2,571
Assigned Eukaryota OTUs	87,912	93,548	13,026	205,617
Assigned Metazoa OTUs	76,057	78,499	11,809	73,060
Metazoa OTUs assigned to genus (unique genera)	24,062 (38)	25,828 (245)	9,831 (69)	28,410 (581)
Metazoa OTUs assigned to species (unique species)	12,084 (114)	23,999 (210)	2,977 (27)	24,209 (75)

The comparative analysis of three databases and related taxonomic assignment methods showed that assigning sequence data to the NCBI reference database resulted in fewer unassigned sequences for both genes. When only metazoan sequences are assessed, the use of the PR2 database resulted in 1,766 (i.e. 7%) less 18S rRNA sequences assigned to genus compared to NCBI, while the BOLD database enabled assignment of 18,579 (i.e. 34%) of COI NCBI assigned sequences. A similar ratio was also observed for sequences assigned to species (Table 1).

### Comparative taxonomic richness and diversity among metabarcoding data

Metabarcoding data yielded up to 76 and 50 (18S rRNA; PR2 database; winter season) taxa per sample plate identified to genus and species level, respectively (Fig. 1).

Notable differences in the number of identified genera or species were observed between markers and seasons, and amongst the three databases (Fig. 1). For example, the number of identified genera or species from 18S rRNA data was higher in winter samples regardless of the database used, whereas an opposite trend was observed in COI (BOLD and NCBI data). Using BOLD, a large number of sequences remained unassigned (Table 1), resulting in fewer than 11 genera and species per sample being identified in summer and winter season. Using COI assigned to NCBI, up to 58 genera could be identified on average while the average number of COI NCBI assigned species was with 25 considerably lower (Fig. 1).

Assessment of the taxonomic diversity among all datasets showed that 80–90% of eukaryote OTUs were assigned to metazoan taxa, with the exception of the COI NCBI dataset (35%; Table 1). The remainder of the latter data were unassigned eukaryotes (51,787 taxa) or belonged to algal groups (65,379). The 18S rRNA data showed a very similar diversity across PR2 and NCBI assignments (Fig. 2), and was mainly comprised of Chordata (more abundant in summer), Nematoda (more abundant in winter) and Bryozoa (consistent across seasons). Cnidaria and Mollusca OTUs were present predominantly in winter. There were more OTUs assigned to Metazoa in the COI NCBI data than in COI BOLD (Table 1). This is reflected in the differences between NCBI and BOLD COI databases in the proportions of the major taxa (Fig. 2). Only NCBI identified the algal groups (Chlorophyta and Ochrophyta), and appeared to identify more Bryozoa and Mollusca. The taxa found using the COI marker gene were more consistent between summer and winter seasons compared to the 18S rRNA marker. Overall, the metabarcoding analyses using COI assigned to NCBI appeared to identify highest diversity of all major taxa compared to the morphological taxonomy and other metabarcoding approaches.

### Detection of putative non-indigenous taxa

**Figure 3:**
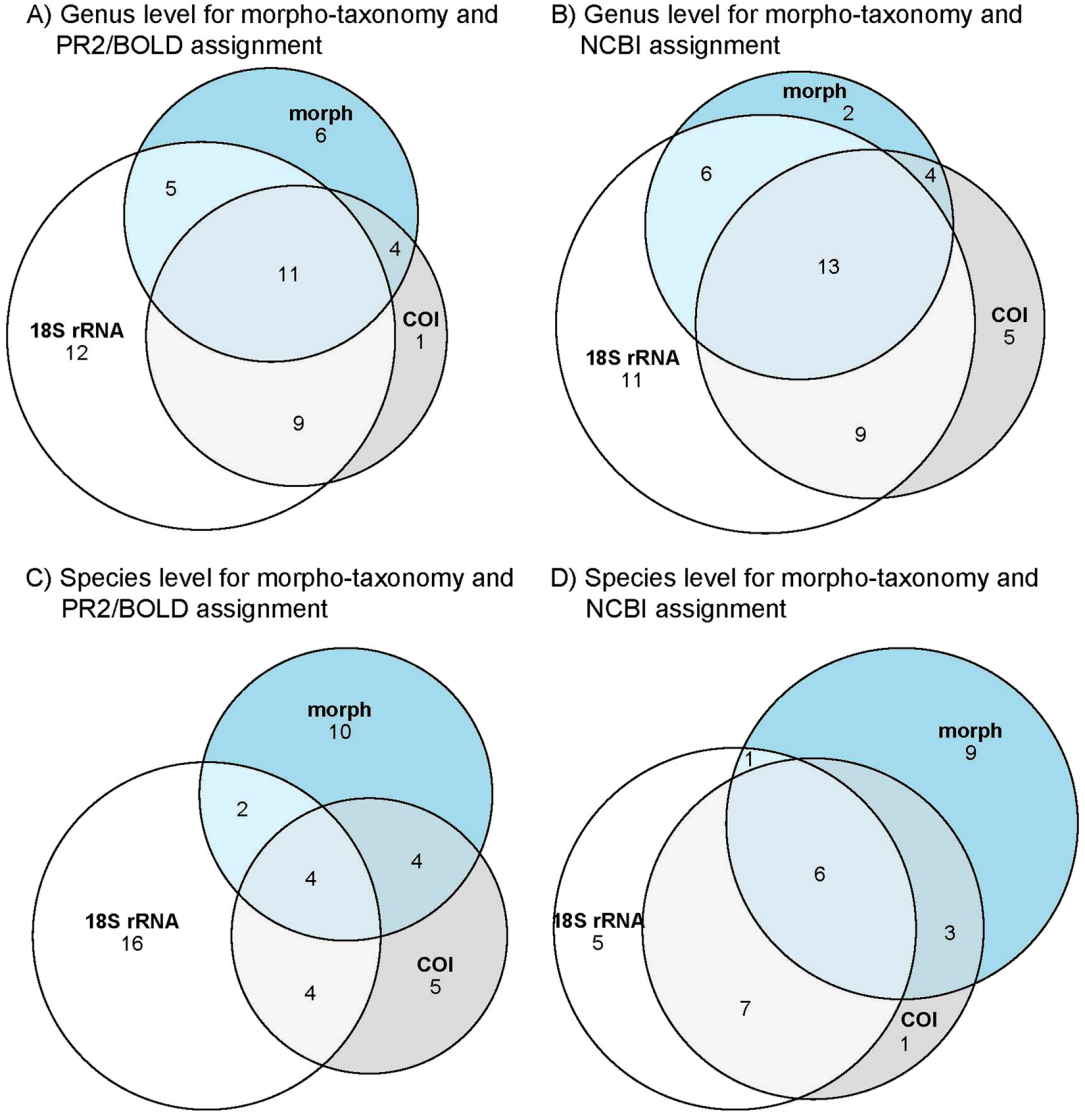
Euler diagrams of non-indigenous taxa identified using the morpho-taxonomy (morph) and metabarcoding approaches, 18S ribosomal RNA (18S rRNA) and *Cytochrome c oxidase Subunit I* (COI). Comparisons at; (**A**) genus level using the PR2 or BOLD databases for taxonomic assignment, (**B**) genus level using NCBI for taxonomic assignment, (**C**) species level using the PR2 or BOLD for taxonomic assignment, and (**D**) species level using NCBI for taxonomic assignment. The size of circles is proportional to the number of genera/species identified by each method.

**Figure 4:**
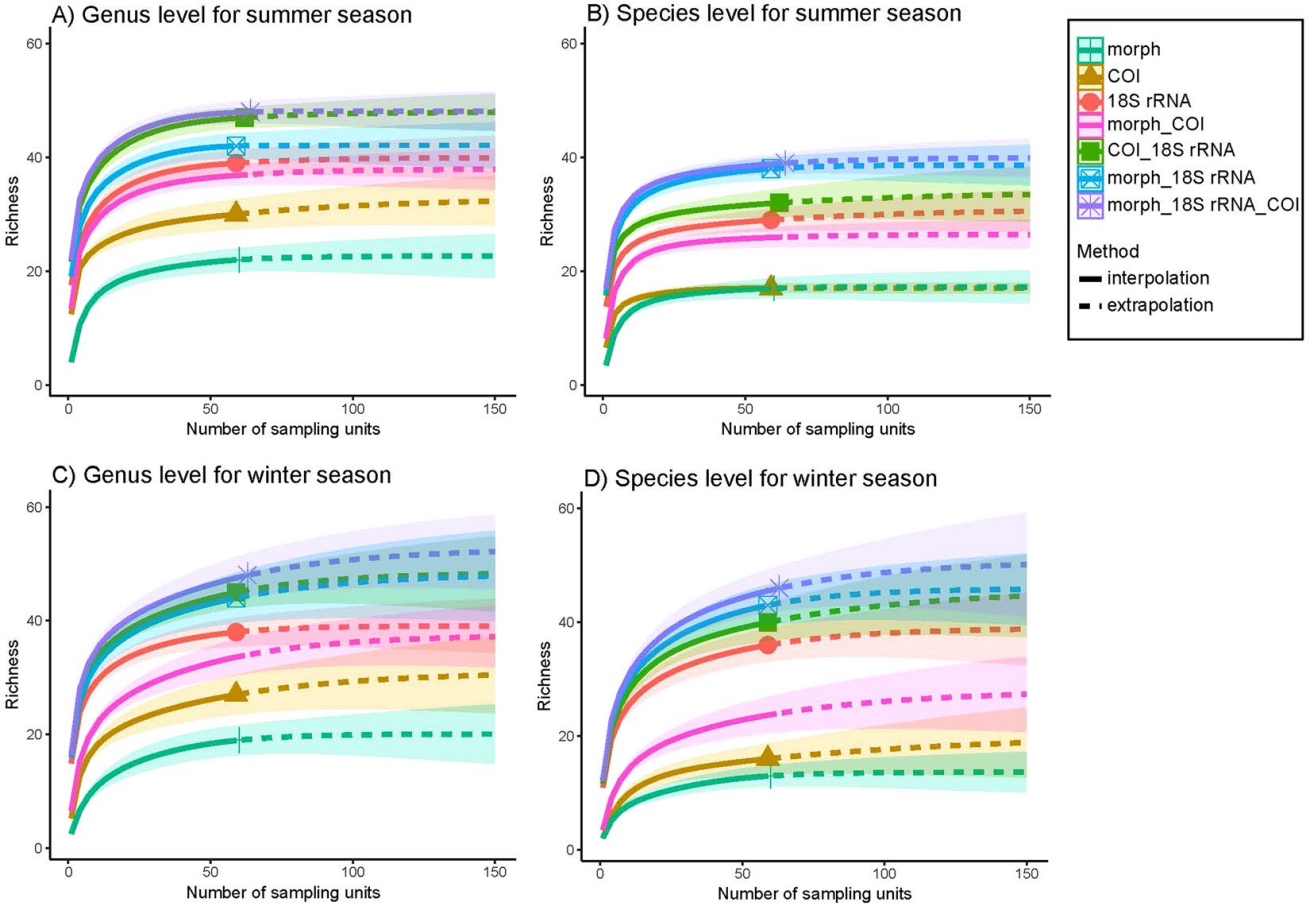
Rarefaction curves of NIS diversity calculated from all detected non-indigenous taxa identified during summer at (**A**) genus and (**B**) species levels and during winter at (**C**) genus and (**D**) species levels. Detected using morpho-taxonomy and metabarcoding (18S ribosomal RNA [18S rRNA] and *Cytochrome c oxidase Subunit I* [COI]) approaches, individually and in combination.

In total, there were 51 NIS genera and 48 NIS species identified across all approaches and assignment methods. The morpho-taxonomic screening for NIS identified three putative non-indigenous genera and seven species (5%/14%, respectively) that were not detected by either molecular markers or the database assignments (Fig. 3; Table S1). These predominantly consisted of bryozoan taxa, e.g. *Bugulina (flabellate)*, *Schizoporella japonica*, *Celleporaria umbonatoidea* and *Tricellaria (inopinata)*. Overall, the dataset using 18S rRNA and PR2 yielded the highest number of unique NIS detections with just two genera but 14 species (3%/29%, respectively). Three genera and three species were uniquely detected using 18S rRNA NCBI such as *Styela clava*. The dataset using COI and BOLD resulted in the detection of one unique species (Figs 3A and 4C), while the dataset using COI and NCBI identified two unique algal NIS (*Striaria* and *Pzeudo-nitzschia;* Fig. 3B; Table S1). A complete list of the potential non-indigenous genera and species detected using morphological and metabarcoding approaches and the different databases is given in Table S1.

A total of 21 genera (e.g. *Arcuatula)* and 11 species (e.g. *Ciona savignyi*, *Arcuatula senhousia*, and *Hydroides elegans*) were detected by at least two of the metabarcoding approaches and database assignments but not by morpho-taxonomy. The combination of morpho-taxonomy with at least one metabarcoding approach led to the detection of 22 non-indigenous genera (e.g. *Bugula*, *Ciona* or *Ectopleura*) and 12 species (e.g. *Ascidiella aspersa*, *Styela plicata* or *Sabella spallanzanii*). Only nine genera (e.g. *Botryllus*, *Styela* and *Hydroides)* and two species (*Botryllus schlosseri* and *Styela plicata*) were detected by all approaches and database assignment methods combined.

Sample-based rarefaction curves for the detection of non-indigenous taxa (Fig. 4) showed that, overall, morpho-taxonomy followed by COI, especially at species level, individually and in combination returned the poorest average per sample diversity, although COI required fewer samples to detect the complete suite of taxa detectable with that method. The 18S rRNA approach was individually the most successful method at detecting diversity with the fewest samples. The different combinations of approaches displayed higher richness of detected NIS than the individual methods. The increase in richness of each combination was approximately equal to the sum of the individual component curves, indicative of the low overlap in detections between all approaches.

In winter, all approaches required more sampling effort than in summer to detect all potential NIS present (Fig. 4C,D). Overall, the combination of all approaches (morph + 18S rRNA + COI) resulted in highest detected richness with the fewest samples, although for summer the combination of 18S rRNA with COI at genus level and 18S rRNA and morpho-taxonomy nearly reached the same efficiency.

## Discussion

The results of the present study add to the growing body of research advocating for the use of parallel morpho-taxonomy and metabarcoding to enhance biodiversity studies and for the sensitive detection of non-indigenous species^5,14,37,56^. This study reinforced the finding that the effectiveness of metabarcoding is strongly influenced by the genetic marker targeted and by the completeness of the sequence databases used for taxonomic assignment^57^. Nevertheless, it is also acknowledged that the use of different parameters or algorithms in the bioinformatic workflow may also influence the final results as observed in e.g. Hatzenbuhler, *et al*.^58^. We assessed the efficiency of two markers (18S rRNA and COI) and three reference databases for taxonomic assignment; PR2 for 18S rRNA, BOLD for COI and NCBI for both characterizing the biodiversity and detecting NIS in marine biofilms/biofouling. The results reveal clear benefits using some approaches, with the greatest success being derived from a combination of approaches.

The bioinformatics pipeline used in this study clustered sequences into OTUs at a 99% similarity threshold and maintained all sequences including singletons. While the voluminous data produced from this approach is resource intensive to analyze, it enhances the likelihood of keeping rare, but potentially important sequences. The removal of singletons is usually employed to eliminate false positives^35^. However, in the context of marine biosecurity surveillance, a false negative could be costly, e.g. the NIS is not detected prior to, or during the early incursion stage when management intervention might be possible. In contrast, a positive result (even if false) can be used to trigger a series of actions to confirm the presence of a NIS including; additional sampling/surveying, further analysis of technical replicates and the use of alternative species-specific molecular approaches such as real-time PCR^59–61^. Nonetheless, the high number of potential false positives observed here and which were likely related to the available bioinformatics workflow, may have serious implications if interpreted in isolation as they might also trigger the deployment of unnecessary and costly investigations. The process of quality control over the interpretation of sequence reads is important in determining its utility. Recent bioinformatics algorithms such as UNOISE^62^ or DADA2 (Divisive Amplicon Denoising Algorithm)^36^, which uses amplicon sequence variants or unique sequences instead of clustering sequences in order to retain as much information as possible, are becoming extremely efficient at denoising data (i.e. removing erroneous sequences). This approach represents an interesting research avenue for analyzing HTS data aimed at detecting rare taxa.

Recent metabarcoding studies have highlighted the advantages of using sequences from several genetic regions to characterize biological communities. This may overcome issues such as limitations in the universality of primers, and extend the different taxonomic levels resolved^63^. In the present study, the COI analysis yielded over twice the number of OTUs compared to the 18S rRNA. A similar result was also reported by Borrell, *et al*.^64^ who investigated the applicability of these two markers to detect NIS in estuaries. The most likely explanation for this result is that the rate of evolution in the COI gene is much faster than in the 18S rRNA and therefore there is greater sequence variability between species^65^. The use of the 99% similarity clustering (described above) contributed to the preservation of this effect^28,35^.

Taxonomy was assigned to OTUs using the PR2 database for 18S rRNA, and BOLD for COI. Additionally, all unique sequences were blasted directly against the NCBI database but using MEGAN’s LCA algorithm for taxonomy assignment when species hits were below 97%. This resulted in marked differences in the number of taxa detected. For the COI data, almost all OTUs remained unassigned when using the BOLD database, however this value was less than 2% when blasted against NCBI. This was largely attributed to the high abundance of bacteria, protists and micro algal taxa in the samples that are not well represented in the BOLD database, which focuses on invertebrates and vertebrates^5,66^. When the COI data was interrogated at genus level, the number of assigned OTUs was also markedly different between the BOLD or NCBI database. The higher Megablast taxonomic assignment rates at genus and species level were reached using 97% species hits and the LCA algorithm for the remaining assignments. The reason was to reach conservative results and to avoid similar false positive detections from both, the UCLUST and Megablast algorithms. However, the NCBI database contains a high number of non-curated data entries many of which come from environmental studies^57^. BLAST searches often return these as the closest match and prevent the algorithm searching for reference sequences which are present in lower abundance but have more definitive taxonomic identity^67^. Customized databases can largely avoid this problem as they contain exclusively curated data and therefore increasing taxon-specific resolution^29,65^. However, the 97% similarity threshold and the LCA algorithm of lower assigned hits was applied to aim for the most conserved taxonomic level in case of several Blast hits of the same similarity threshold but different taxonomy^67^.

A similar pattern was observed for 18S rRNA data. About 8% of OTUs were unassigned using the PR2 database whereas this reduced to about 2% when using the NCBI database. When only Eukaryotes and Metazoans were considered, the assignment using either database was comparable^31^. When taxonomy was assigned at the genus level there were up to six times more uniquely assigned taxa in NCBI compared to PR2 while the overall genus assignments showed very little difference (7%); this again is likely related to the high abundance of non-curated data-entries or not further determined ‘sp.’ species in NCBI and the Megablast search on the highly conserved 18S region that can reveal several hits of the same similarity threshold.

The morpho-taxonomic approach employed in the present study resulted in the lowest rate of identified taxa, a result which has been shown in many other studies^23,57,68^. A plethora of factors likely contribute to the low detection rate of taxa including; an inability to detect micro-organisms, cryptic taxa, larval stages, and the presence of extracellular DNA which is only detected using molecular techniques^39^. Morpho-taxonomy, however, is still the technique that results in the fewest false positive errors. Additionally, in the present study a number of taxa, most notably Bryozoan taxa, were detected using morphology but not metabarcoding. A search of the customized databases (PR2 and BOLD) used in the present study revealed that there were no representative sequences for most bryozoans detected by the morphological approach. However, NCBI has several entries for all these taxa (18S rRNA and COI regions). This suggests that amplification of bryozoan taxa failed possibly due to incompatible primer binding sites, primer affinity or degraded DNA within the bulk samples. Lejzerowicz, *et al*.^15^ targeted the same V4 18S rRNA region and also failed to identify bryozoans that were morphologically detected. Berry, *et al*.^57^ identified a similar issue with the COI mini-barcode, failing to detect most mollusc taxa. In the present study, the non-detection of some species using metabarcoding could also be due to the highly diverse communities found in the biofouling samples, as sensitivity and accuracy of metabarcoding has been shown to be influenced by taxon composition and abundance^58,60^. Collectively, the present results and those from similar studies indicate that the greatest and most complete biodiversity inventories will be obtained using a combined metabarcoding and morphological approach, which has the additional benefit of enabling cross-verification of specific detections, such as rare or invasive species of interest^58^.

In addition to exploring biodiversity, this study aimed to evaluate each approach for its ability to identify potential NIS. Considering only the molecular approaches, the lowest NIS detection was obtained when taxonomic assignment was undertaken using NCBI and the LCA algorithm alone. To avoid the low assignment rates using the LCA algorithm, we combined the taxonomical assignment for potential NIS retrieving direct best hits of at least 97% identity. With the exception of five algal genera uniquely identified using NCBI for 18S rRNA and COI, all other NIS were also identified when assignment was undertaken using the PR2 or BOLD databases (Table S1). It is usually anticipated that taxonomic resolution will be low when targeting the 18S rRNA compared to COI^65^. However, in this study the greatest detection of potential non-indigenous species (*n* = 14) occurred when using the 18S rRNA and PR2 database. These results should be treated with some caution due to the highly conserved nature of 18S that might result in assignment of an OTU to a closely related genus or species using less stringent algorithms such as UCLUST. However, a less stringent approach is still valid for flagging potential NIS. We recommend interrogating the flagged data more closely to assess the validity of the positive results prior to initiating further actions. For example, *Asterias forbesi* was identified only with 18S rRNA. A manual blast assigned it to the closely related species *Asterias amurensis*, the highly undesirable seastar, which is blacklisted in the World Register of Introduced Marine Species (WRIMS), and is not yet known to occur in New Zealand. However, these uncertain observations likely represent a false positive detection. The sequence was only present in low abundance (8 reads in 20 samples) and when using the NCBI database and the LCA algorithm for the taxonomy assignment, these sequences were assigned taxonomically only to the family level, i.e. ‘Asteroidea’. It is most likely that this detection is not *A*. *forbesi*, but that of a closely related taxon whose sequence is absent from current databases. Even in this case, this result is valuable in the marine surveillance context as it might be effectively used for triggering the application of a species-specific *A*. *amurensis* diagnostic test (e.g. real-time PCR) possibly confirming the presence of this unwanted organism in these samples. A further example of the challenges associated with NIS detection using metabarcoding is the invasive marine fanworm *Sabella spallanzanii*, which was detected by three approaches (18S rRNA [PR2], COI [BOLD] and morpho-taxonomic data) but not when taxonomy was assigned using NCBI. When the NCBI database was used, taxonomy could not be assigned below the genus level i.e. ‘Sabella’. Overall, these examples highlight the on-going need for the development of regionally specific reference databases, and the requirement for additional markers that provide accurate species-specific resolution. By contrast, both molecular markers identified the presence of the invasive taxa *Arcuatula senhousia*, *Ciona savignyi* and *Amathia gracilis*. However, they were not identified using morpho-taxonomy. As noted above this is most likely because they were in their larval stage, the metabarcoding detected extracellular DNA, or a closely related species was present and taxonomy was incorrectly assigned (e.g. *Ciona intestinalis*).

Rarefaction curves were used to explore how including additional approaches enhanced the detection of NIS. When the sequencing depth was kept similar among molecular data, the 18S rRNA gene resulted in the highest NIS detection, while metabarcoding of the COI and morpho-taxonomy performed equally. In the present study, 25% of NIS taxa identified at species level were present in both morphological and metabarcoding datasets, a result which is similar to the 20% shared by both methods in Lejzerowicz, *et al*.^15^. Eighty percent of NIS detections did not overlap between morphological and metabarcoding approaches, highlighting how using these approaches in parallel may enhance the detection of NIS. Interestingly, the number of NIS detected using metabarcoding did not reach diversity saturation in the winter samples. There is the possibility that some of the variation observed in this study between winter and summer datasets resulted from using different sampling techniques (swabbing versus scraping). It is also likely that the lower number of dominating macro-organisms in the winter settlement plates favored the colonization of a more diverse microbial assemblage among biofouling meta-communities^39^.

## Conclusions

Bioinformatic pipelines are continually evolving and careful consideration must be given to the methodology applied, which should be based on the specific aims of the study. In the present investigation, we employed stringent clustering (99%) without denoising steps. Although this likely maintains some sequencing errors which may result in inflation of biodiversity values, and potentially the false positive detection of NIS, it increases the likelihood of NIS detection and the initiation of more stringent detection procedures. Further important considerations are the target gene and reference database used for taxonomic assignment. In this study, the taxonomy of the communities and levels of resolution varied markedly according to the gene and database used. Our results highlight the benefits of including at least two molecular markers when attempting to obtain a detailed overview of the diversity of highly complex marine biofouling communities. The results also highlight biases in each of the different databases and identification algorithms. Each method showed both strengths and weaknesses, suggesting that the most accurate results come from the use of a combination of methods. This allows for cross-validation and maximizes the coverage of NIS. While new markers are continually being developed and databases improved, caution should be applied when interpreting metabarcoding data due to primer bias and incomplete or incorrectly annotated references databases. This was evidenced in this study by the absence of most Bryozoa from the metabarcoding data, despite being highly abundant in morphological data, and the likely false detection such as the differing *Asterias* assignments. We therefore advocate for the use of metabarcoding as a screening method when aiming to detect NIS, with positive detections triggering the application of more targeted molecular methods or in-depth morphological analysis. The inclusion of data from the metabarcoding of two markers and morpho-taxonomy resulted in the highest number of potential NIS detections, suggesting that combining these methods will enhance marine biosecurity surveillance.

## Electronic supplementary material

Supplementary information
