# Supplementary material for: Combining morpho-taxonomy and metabarcoding enhances the detection of non-indigenous marine pests in biofouling communities
Source: Sci Rep. 2018 Nov 2;8:16290. doi: 10.1038/s41598-018-34541-1 (PMC6215007; doi:10.1038/s41598-018-34541-1)
Supplement: Supplementary file 1 — Supplementary information [file 41598_2018_34541_MOESM1_ESM.docx]

**Supplement:**

Combining morpho-taxonomy and metabarcoding enhances the detection of non-indigenous marine pests in biofouling communities

Ulla von Ammon^a,b*^, Susanna A Wood^a^, Olivier Laroche^a,b^, Anastasija Zaiko^a,b^, Leigh Tait^c^, Shane Lavery^b,d^, Graeme J. Inglis^c^, Xavier Pochon^a,d^

*^a^Environmental Technologies, Coastal and Freshwater Group, Cawthron Institute, Private Bag 2, Nelson 7042, New Zealand;*

*^b^School of Biological Sciences, University of Auckland, Private Bag 349, Warkworth 0941, New Zealand;*

*^c^National Institute of Water & Atmospheric Research Ltd, PO Box 8602, Riccarton, Christchurch 8440, New Zealand*

*^d^Institute of Marine Science, University of Auckland, Private Bag 349, Warkworth 0941, New Zealand*

Table S1: List of New Zealand non-indigenous taxa identified in this study. Non-indigenous status defined by reference to the New Zealand Organisms Register ^51^ and Marine Biosecurity Porthole^50^. NIS detections are shown at genus and species levels for each approach: morpho-taxonomy, 18S ribosomal rRNA (18S rRNA) assigned against the Protist Ribosomal Reference database (PR2) and the National Centre for Biotechnology Information (NCBI), and *Cytochrome c oxidase Subunit I* (COI) assigned against the Barcode of Life Database (BOLD) and NCBI.

|  | **genus** | | | | |  | **species** | | | | |
| --- | --- | --- | --- | --- | --- | --- | --- | --- | --- | --- | --- |
|  | **18S rRNA** | | **COI** | | **morph** |  | **18S rRNA** | | **COI** | | **morph** |
|  | PR2 | NCBI | BOLD | NCBI |  |  | PR2 | NCBI | BOLD | NCBI |  |
| ***Alexandrium**** | x | x |  |  |  | ***Alexandrium minutum**** | x |  |  |  |  |
| ***Amathia*** | x | x | x | x | x | ***Amathia verticillata*** |  | x | x | x | x |
|  |  |  |  |  |  | ***Amathia gracilis*** | x |  | x |  |  |
|  |  |  |  |  |  | ***Amathia imbricata*** | x |  |  |  |  |
| ***Antithamnionella***** | x | x |  |  |  |  |  |  |  |  |  |
| ***Amphilectus*** |  |  |  |  | x | ***Amphilectus fucorum*** |  |  |  |  | x |
| ***Arcuatula*** | x | x | x | x |  | ***Arcuatula senhousia*** | x | x | x | x |  |
| ***Ascidiella*** | x | x | x | x | x | ***Ascidiella aspersa*** |  | x | x | x | x |
| ***Asterias*** | x | x |  |  |  | ***Asterias forbesi****** | x | x |  |  |  |
| ***Avicennia***** | x | x |  |  |  |  |  |  |  |  |  |
| ***Balanus***** |  |  | x | x | x |  |  |  |  |  |  |
| ***Bonamia*** | x | x |  |  |  | ***Bonamia sp.*** | x |  |  |  |  |
| ***Botrylloides*** |  | x | x | x | x | ***Botrylloides leachii*** |  | x | x | x | x |
| ***Botryllus*** | x | x | x | x | x | ***Botryllus schlosseri*** | x | x | x | x | x |
| ***Bougainvillia**** | x | x | x | x |  |  |  |  |  |  |  |
| ***Bugula*** | x |  | x | x | x | ***Bugula neritina*** |  |  | x | x | x |
| ***Bugulina*** |  |  |  |  | x | ***Bugulina flabellata*** |  |  |  |  | x |
| ***Celleporaria***** |  |  |  | x | x | ***Celleporaria nodulosa*** |  |  |  | x | x |
|  |  |  |  |  |  | ***Celleporaria umbonatoidea*** |  |  |  |  | x |
| ***Chaetomorpha*** | x | x |  |  | x | ***Chaetomorpha crassa*** | x |  |  |  |  |
|  |  |  |  |  |  | ***Chaetomorpha linum****** | x |  |  |  |  |
| ***Chaetopterus**** | x | x |  |  |  |  |  |  |  |  |  |
| ***Ciona*** | x | x | x | x | x | ***Ciona intestinalis*** | x |  |  |  | x |
|  |  |  |  |  |  | ***Ciona savignyi*** | x | x | x | x |  |
| ***Clathria**** | x |  |  |  |  | ***Clathria prolifera**** | x |  |  |  |  |
| ***Clytia* ***** | x | x | x | x |  |  |  |  |  |  |  |
| ***Colpomenia***** |  |  |  | x | x |  |  |  |  |  |  |
| ***Corella*** | x | x |  | x | x | ***Corella eumyota**** | x | x |  | x | x |
| ***Corophium**** | x | x |  |  |  |  |  |  |  |  |  |
| ***Cryptosula*** |  | x |  |  |  | ***Cryptosula pallasiana*** |  | x |  |  |  |
| ***Diplosoma*** | x | x | x | x |  | ***Diplosoma ooru****** | x |  |  |  |  |
| ***Dorvillea* ***** | x |  |  |  |  |  |  |  |  |  |  |
| ***Ecteinascidia****** | x | x |  |  |  |  |  |  |  |  |  |
| ***Ectopleura*** | x | x | x | x | x | ***Ectopleura crocea*** |  | x | x | x |  |
|  |  |  |  |  |  | ***Ectopleura dumortierii****** | x |  |  |  |  |
|  |  |  |  |  |  | ***Ectopleura larynx*** | x |  |  |  |  |
| ***Electra***** | x |  |  | x |  |  |  |  |  |  |  |
| ***Gymnodinium*** | x | x |  | x |  |  |  |  |  |  |  |
| ***Halecium***** | x | x |  | x |  | ***Halecium muricatum****** | x |  |  |  |  |
| ***Hydroides*** | x | x | x | x | x | ***Hydroides elegans*** | x | x | x | x |  |
| ***Leucosolenia ***** | x | x |  |  | x |  |  |  |  |  |  |
| ***Limaria***** |  | x |  |  |  |  |  |  |  |  |  |
| ***Magallana*** | x | x | x | x | x | ***Magallana gigas*** | x |  | x | x | x |
| ***Microcosmus***** | x | x | x | x |  | ***Microcosmus squamiger**** |  | x | x | x |  |
| ***Molgula***** | x | x |  |  | x | ***Molgula complanata****** | x |  |  |  |  |
|  |  |  |  |  |  | ***Molgula manhattensis*** |  | x |  |  | x |
|  |  |  |  |  |  |  |  |  |  |  |  |
| ***Mya* ****** | x | x |  |  |  |  |  |  |  |  |  |
| ***Neosiphonia*** | x |  | x | x |  | ***Neosiphonia harveyi*** |  |  | x |  |  |
| ***Obelia*** | x | x | x | x |  | ***Obelia geniculata*** | x | x |  |  |  |
| ***Polydora*** | x | x | x | x |  | ***Polydora haswelli*** | x |  |  |  |  |
|  |  |  |  |  |  | ***Polydora hoplura*** | x |  |  |  |  |
| ***Pseudo-nitzschia**** |  |  |  | x |  |  |  |  |  |  |  |
| ***Sabella*** | x | x | x | x | x | ***Sabella pavonina****** |  | x | x | x |  |
|  |  |  |  |  |  | ***Sabella spallanzanii*** | x |  | x |  | x |
| ***Schizoporella*** |  | x |  |  | x | ***Schizoporella japonica*** |  |  |  |  | x |
| ***Striaria*** |  |  |  | x |  | ***Striaria attentuata*** |  |  |  | x |  |
| ***Styela*** | x | x | x | x | x | ***Styela plicata**** | x | x | x | x | x |
|  |  |  |  |  |  | ***Styela clava*** |  | x |  |  |  |
| ***Symplegma*** | x | x |  |  | x | ***Symplegma viride****** | x |  |  |  |  |
|  |  |  |  |  |  | ***Symplegma brakenhielmi*** |  |  |  |  | x |
| ***Tricellaria*** |  |  |  |  | x | ***Tricellaria inopinata*** |  |  |  |  | x |
| ***Ulva*** |  | x |  |  |  | ***Ulva intestinalis*** |  | x |  |  |  |
| ***Watersipora*** |  | x | x | x | x | ***Watersipora subatra*** |  |  |  |  | x |
|  |  |  |  |  |  | ***Watersipora subtorquata*** |  | x | x | x |  |
| **Σ 51** | 37 | 38 | 21 | 29 | 24 | **48** | 26 | 19 | 17 | 17 | 19 |

** indeterminate/cryptogenic taxa for New Zealand but known to be non-indigenous elsewhere*

***genus contains indigenous and non-indigenous species*

****not previously mentioned in NZ*

Table S2: List of non-indigenous species (NIS) considered in this study (Table S1) and their available reference sequences per database. Database versions were pr2_gb203_version_4.5 for PR2, taxo_bold_01_2018 and Midori_unique_20180221 for BOLD and NCBI updated version in May 2018. Reference sequences for the different taxa were indicated with ‘x’ when available and ‘o’ when missing.

| **genus** | **PR2** | **BOLD** | **NCBI**  **(18S)** | **NCBI**  **(COI)** | **species** | **PR2** | **BOLD** | **NCBI**  **(18S)** | **NCBI**  **(COI)** |
| --- | --- | --- | --- | --- | --- | --- | --- | --- | --- |
| *Alexandrium* | x | o | x | x | *Alexandrium minutum* | x | o | x | x |
| *Amathia*  *(Bowerbankia,*  *Zoobotryon)* | x | x | x | x | *Amathia verticillata* | x | x | x | x |
|  |  |  |  |  | *Amathia gracilis* | x | x | o | x |
|  |  |  |  |  | *Amathia imbricata* | x | o | x | x |
| *Antithamnionella* | x | o | x | x |  |  |  |  |  |
| *Amphilectus* | x | o | x | x | *Amphilectus fucorum* | x | o | x | x |
| *Arcuatula*  *(Musculista)* | x | x | x | x | *Arcuatula senhousia* | x | x | x | x |
| *Ascidiella* | x | x | x | x | *Ascidiella aspersa* | x | x | x | x |
| *Asterias* | x | x | x | x | *Asterias forbesi* | x | x | x | x |
| *Avicennia* | x | o | x | o |  |  |  |  |  |
| *Balanus* | x | x | x | x |  |  |  |  |  |
| *Bonamia* | x | o | x | o | *Bonamia sp.* | x | o | x | o |
| *Botrylloides* | x | x | x | x | *Botrylloides leachii* | o | x | x | x |
| *Botryllus* | x | x | x | x | *Botryllus schlosseri* | x | x | x | x |
| *Bougainvillia* | x | x | x | x |  |  |  |  |  |
| *Bugula* | x | x | x | x | *Bugula neritina* | x | x | x | x |
| *Bugulina* | o | o | o | x | *Bugulina flabellata* | o | o | o | o |
| *Celleporaria* | x | o | x | x | *Celleporaria nodulosa* | o | o | x | x |
|  |  |  |  |  | *Celleporaria umbonatoidea* | o | o | o | o |
| *Chaetomorpha* | x | o | x | o | *Chaetomorpha crassa* | x | o | x | o |
|  |  |  |  |  | *Chaetomorpha linum* | x | o | x | o |
| *Chaetopterus* | x | o | x | x |  |  |  |  |  |
| *Ciona* | x | x | x | x | *Ciona intestinalis* | x | x | x | x |
|  |  |  | x | x | *Ciona savignyi* | x | x | x | x |
| *Clathria* | x | o | x | x | *Clathria prolifera* | x | o | x | x |
| *Clytia* | x | x | x | x |  |  |  |  |  |
| *Colpomenia* | x | o | x | x |  |  |  |  |  |
| *Corella* | x | x | x | x | *Corella eumyota* | x | x | x | x |
| *Corophium* | x | x | x | x |  |  |  |  |  |
| *Cryptosula* | x | o | x | x | *Cryptosula pallasiana* | x | o | x | x |
| *Diplosoma* | x | x | x | x | *Diplosoma ooru* | x | o | x | o |
| *Dorvillea* | x | x | x | x |  |  |  |  |  |
| *Ecteinascidia* | x | x | x | x |  |  |  |  |  |
| *Ectopleura* | x | x | x | x | *Ectopleura crocea* | x | x | x | x |
|  |  |  |  |  | *Ectopleura dumortierii* | x | x | x | x |
|  |  |  |  |  | *Ectopleura larynx* | x | x | x | x |
| *Electra* | x | o | x | x |  |  |  |  |  |
| *Gymnodinium* | x | o | x | x |  |  |  |  |  |
| *Halecium* | x | x | x | x | *Halecium muricatum* | x | x | x | o |
| *Hydroides* | x | x | x | x | *Hydroides elegans* | x | x | x | x |
| *Leucosolenia* | x | o | x | o |  |  |  |  |  |
| *Limaria* | x | x | x | x |  |  |  |  |  |
| *Magallana* | x | x | x | x | *Magallana (Crassostrea) gigas* | x | x | x | x |
| *Microcosmus* | x | x | x | x | *Microcosmus squamiger* | x | x | x | x |
| *Molgula* | x | x | x | x | *Molgula complanata* | x | o | x | o |
|  |  |  |  |  | *Molgula manhattensis* | x | x | x | x |
| *Mya* | x | x | x | x |  |  |  |  |  |
| *Neosiphonia* | x | x | x | x | *Neosiphonia harveyi* | x | x | x | x |
| *Obelia* | x | x | x | x | *Obelia geniculata* | x | x | x | x |
| *Polydora* | x | x | x | x | *Polydora haswelli* | x | x | o | x |
|  |  |  |  |  | *Polydora hoplura* | x | o | x | x |
| *Pseudo-*  *nitzschia* | x | o | x | x |  |  |  |  |  |
| *Sabella* | x | x | x | x | *Sabella pavonina* | x | x | x | x |
|  |  |  | x | x | *Sabella spallanzanii* | x | x | x | x |
| *Schizoporella* | x | o | x | x | *Schizoporella japonica* | o | o | o | o |
| *Striaria* | o | o | o | x | *Striaria attentuata* | o | o | o | o |
| *Styela* | x | x | x | x | *Styela plicata* | x | x | x | x |
|  |  |  | x | x | *Styela clava* | x | x | x | x |
| *Symplegma* | x | o | x | x | *Symplegma viride* | x | o | x | o |
|  |  |  |  |  | *Symplegma brakenhielmi* | o | o | o | x |
| *Tricellaria* | o | o | x | x | *Tricellaria inopinata* | o | o | o | x |
| *Ulva* | x | o | x | o | *Ulva intestinalis* | x | o | x | o |
| *Watersipora* | x | x | x | x | *Watersipora subatra* | o | o | o | o |
|  |  |  |  |  | *Watersipora subtorquata* | x | x | x | x |

Table S3: Overview of OTUs assigned to each species for 18S rRNA and COI datasets. Singletons = OTUs with only one read.

|  | 18S rRNA | COI |
| --- | --- | --- |
| Number of OTUs assigned to a species | 546 | 106 |
| Number of species represented by only 1 OTU | 221 | 52 |
| Number of species represented by a singleton | 36 | 26 |
| Median number of OTUs per species | 4 | 36.5 |
| Median of singletons | 1 | 25 |
